# Satellite DNAs—From Localized to Highly Dispersed Genome Components

**DOI:** 10.3390/genes14030742

**Published:** 2023-03-17

**Authors:** Eva Šatović-Vukšić, Miroslav Plohl

**Affiliations:** Division of Molecular Biology, Ruđer Bošković Institute, 10000 Zagreb, Croatia

**Keywords:** satellite DNA, repetitive DNA, transposable element, satellitome, heterochromatin, euchromatin

## Abstract

According to the established classical view, satellite DNAs are defined as abundant non-coding DNA sequences repeated in tandem that build long arrays located in heterochromatin. Advances in sequencing methodologies and development of specialized bioinformatics tools enabled defining a collection of all repetitive DNAs and satellite DNAs in a genome, the repeatome and the satellitome, respectively, as well as their reliable annotation on sequenced genomes. Supported by various non-model species included in recent studies, the patterns of satellite DNAs and satellitomes as a whole showed much more diversity and complexity than initially thought. Differences are not only in number and abundance of satellite DNAs but also in their distribution across the genome, array length, interspersion patterns, association with transposable elements, localization in heterochromatin and/or in euchromatin. In this review, we compare characteristic organizational features of satellite DNAs and satellitomes across different animal and plant species in order to summarize organizational forms and evolutionary processes that may lead to satellitomes’ diversity and revisit some basic notions regarding repetitive DNA landscapes in genomes.

## 1. Introduction

Eukaryotic genomes are highly enriched with non-protein-coding repetitive sequences, which form the largest but still the least understood component of genomic DNA. Two main classes have been traditionally considered, repetitive sequences organized as tandem repeats and those interspersed throughout the genome [1,2]. Because of difficulties in sequencing and assembly, they are often known as the “dark matter of genomes”, which, in outputs of genome projects, became represented more accurately only after the advent of long-range sequencing and introduction of specialized bioinformatics tools [3,4,5,6].

Satellite DNA (SatDNA) sequences, in the traditional view, appear as megabase-long arrays of many thousands of highly similar head-to-tail tandemly repeated units (monomers) localized in heterochromatic chromosomal segments [7,8,9,10]. They were discovered in experiments of density gradient centrifugation in which an accompanying “satellite” band appeared due to differences in nucleotide composition with regard to the bulk genomic DNA [11,12]. This generic name continued to be used, irrespective of the method of detection or characteristics of sequences repeated in tandem [8,10]. The other class is made up of interspersed repeats formed as a result of transposition processes, introducing transposable elements (TEs) into new locations, changing, in this process, genome structure, adaptability and evolution [13,14,15,16]. Both satDNAs and TEs are considered crucial builders of every eukaryotic genome and drivers of evolution [1,2,17,18,19,20]. 

It is, thus, more and more evident that a complete understanding of every eukaryotic genome is possible with only a detailed insight into its repetitive fraction. This is not an easy task, and, in general, we are still far from full comprehension regarding repetitive DNA genomics and their diversity (for example, [21]). Nevertheless, the burst of methodological approaches in recent years significantly accelerated the accumulation of previously inaccessible data, broadened the number of attended species and detected repetitive DNA families, changing the views and established concepts (reviewed in [6,8,10,18,22,23]). The result are satellitomes and repeatomes, defined as collections of all satDNAs and repetitive DNAs, respectively [24,25]. Furthermore, the third-generation sequencing pipelines produce several hundred kb-long high-quality reads, including satDNAs [4,26]. While short-read-based approaches are limited in assessing the exact arrangement of repeats in the genome, the later methodology forwarded chromosome-level assemblies and enabled detailed insights into large repetitive regions (for example, [27,28,29,30,31,32,33,34]). 

A growing number of studied species and data accumulated on the genomics scale emphasized extreme variations in the general architecture of satDNAs, not only in the number and abundance of families but also in their genomic distribution, heterochromatin/euchromatin localization, array length and association with TEs. Differences indicate conceptual specificities in repetitive DNA organization, in particular, taxonomic groups and the need to expand the number of model systems [10]. In the present review, we compare data in the light of different organizational forms of satDNAs and repetitive DNAs, in general, in an attempt to summarize conceptual differences in repetitive DNA landscapes and evolutionary processes that may cause their diversity.

## 2. Functional Importance of Satellite DNAs and Partnership with TEs

Data accumulated in the last few decades significantly changed the classical notion about satDNA sequences as a non-functional ballast deposited in heterochromatin to significant contributors in defining chromosomal architecture, function and evolution [18,23,35,36,37]. They are the most frequent DNA components in centromeres and contribute to the essential process of assembly of centromeric chromatin [33,38,39]. SatDNAs are involved in meiotic drive and segregation of chromosomes [40,41], and their evolution can trigger reproductive isolation and speciation [42]. Pericentromeric satDNAs also contribute to higher-level organization of nucleus and in preserving genome integrity [43]. Not only as a structural genomic component but also long-time neglected transcription of satDNAs showed its functional importance, such as in the formation and maintenance of heterochromatin itself, in defining centromere identity and preserving genome stability (reviewed in [5,37,44]). Misregulation of satDNA expression can lead to various abnormalities in the genomic architecture, chromosome segregation and gametogenesis. Changes in copy number of satDNAs and their transcription rates may be associated with stress, environmental adaptations and pathological states, such as oncogenic transformation [18,45,46,47,48,49,50,51]. 

An increasing number of reports show that satDNAs and TEs are tightly connected in many different ways, and TEs, in general, were proposed to facilitate the dispersal of satDNA repeats (reviewed in [17,20,52,53]). Tandemization of TEs or their parts can be accomplished through different mechanisms, resulting in arrays of repeats characteristic for a satDNA [54]. Of particular interest are Helitrons and related DNA transposons, widespread in plants and animals, which use rolling-circle replication in their spread [55,56]. These elements can incorporate variable numbers of tandem repeats (usually up to 10) as their central structural components that can be found also as standalone, as typical satDNA arrays [57,58,59,60,61,62]. Rolling-circle replication, therefore, stands out as an efficient way to distribute and amplify tandem repeats throughout the genome [63,64]. Scalvenzi and Pollet [65] proposed a model of possible directions in the life of the TE-incorporated tandem repeats. They suggested that satDNA sequences can be captured by a TE, followed by amplification of tandem repeats within. The transposition of elements containing tandem repeats continues; however, as the number of repeats within the TE is increasing, the transposition rate of the TE is decreasing. In parallel, with the growing number of monomers and the expansion of tandem repeats, recombination rates start to increase. Consequently, TE-incorporated tandem repeats can give rise to the classical satDNA arrays, which are further spread and maintained by unequal crossover and other mechanisms of nonreciprocal transfer [8,9,66]. 

The number of tandemly repeated monomers within TEs is highly variable and, for example, can reach ~90 in Helitrons of the Pacific oyster *Crassostrea gigas* [62]. It must be noted that such hybrid structures, when abundant, can be serious obstacles in classifications of repetitive sequences and the cause of a large fraction of unclassified or misclassified repeats in screening genome project outputs, or in analyses based on short NGS reads [67]. Such classification problems could be resolved by manual adjustments, assignments on segments obtained by third-generation sequencing, and by constant improvements in bioinformatics tools and databases (for example, [4,6]).

## 3. Diversity, Homogeneity and Evolution of satDNAs

SatDNAs represent an extremely diverse group of sequences, as probably almost any genomic segment can be amplified into arrays of tandem repeats. They differ in nucleotide sequence, AT content, DNA structural features (such as sequence-induced DNA curvature and dyad structures), monomer length and complexity, copy number, array length, epigenetic modifications, chromatin state and chromosomal distribution. The various features of satDNA monomer sequences, their epigenetic modifications and interactions with protein components in chromatin have been extensively reviewed [8,23,66,68]. 

Two major common characteristics of sequences repeated in tandem are low sequence variability in repeat units and extreme variability in copy numbers [7,8,9,22,23,66,69]. According to the concept of concerted evolution, monomers in arrays of satDNA evolve together, and low sequence variability is maintained among them. This is because mutations occurring in monomers are homogenized (spread or eliminated) within arrays and in the genome by diverse mechanisms of non-reciprocal sequence exchanges (unequal crossover, gene conversion, mechanisms related to transposition and similar) and fixed among reproductively linked individuals [70,71,72,73]. The process, known as molecular drive, is assumed to be stochastic in nature [74,75,76]. Consequently, while homogeneity of satDNA is preserved within species, its DNA sequence in separated groups of individuals should diverge rapidly, as different mutations are homogenized and fixed in monomers of each group, forming species-specific variants. Depending on the rate, such gradual accumulation of divergences in satDNA sequences can mirror phylogeny at different levels [9,77,78,79]. 

Another model proposes that mutations accumulate among monomers of satDNA, slowing down and ultimately disabling mechanisms of sequence homogenization, leading to divergent (non-concerted) evolution until the deterioration of arrays as repetitive structures. This assumption has been integrated into the life cycle model of satDNA evolution [80], proposing also that in the same time, some divergent monomer (or an unrelated sequence) can be amplified and form a novel (sub)family of highly homogeneous tandem repeats. In support, computational models and experimental analyses revealed that homogenization mechanisms are less efficient at array ends, and that diverged monomers at array ends can be a source of new satDNAs [70,81,82]. Recent high-throughput comparative analysis of grasshopper satellitomes is in agreement with the idea about cycles of occasional amplification of a monomer variant into new homogeneous satDNA arrays, followed by an increase in sequence variability among monomers. According to this concept, younger satDNAs are more homogeneous, showing leptokurtic distribution of monomer sequence variability compared to the consensus sequence [83]. 

## 4. Genomic Content or How Many satDNAs Are in the Genome

Even early studies anticipated that more than one or even many satDNAs, extremely different in copy number, usually exist in the genome. Some satDNAs can build 30% or even more genomic DNA while, in the same time, the genome can hold far less abundant tandem repeats, even <0.1%, easily overlooked with early methodologies [8,66,84]. The same mechanisms of non-reciprocal exchanges leading to concerted evolution are responsible for extensive copy number alterations in arrays of sequences repeated in tandem, abruptly changing their genomic content. Consequently, in the process of speciation, one highly abundant satDNA can contract to low copies, while some low-copy satDNA can expand and become a major satDNA. A set of satDNAs inherited from a common ancestor is the basis of the library model [85]. According to this scenario, copy-number changes alone can be sufficient to explain the rapid evolution of the satDNA landscape and replacement of most abundant satDNA(s) in some species. In this regard, the basic question to be addressed is to characterize the entire satellitome, i.e., to answer how many satDNAs are in the genome and what are their basic features.

Although different approaches exist [6], the recent widely used strategy in detecting the entirety of repetitive DNAs is by clustering next-generation sequencing (NGS) short reads obtained at low genome coverage in order to detect only sequences represented in multiple copies, without the need for the genome assembly. Repetitive sequences are further classified based on graphical constructs, which reveal characteristic circular-shaped forms for clusters of sequences repeated in tandem, classified as satDNAs [24,86,87,88,89,90]. The above-mentioned approaches are used to define satellitomes and repeatomes in an exponentially growing number of studies, oriented to plant and animal species (Table 1), making an extremely valuable contribution towards the comprehension of the repetitive genome landscapes and their evolution. In experimental verification of the obtained results, fluorescence in situ hybridization (FISH) of prophase/metaphase chromosomes and/or chromatin fibers is a valuable method of choice to explore the distribution of satDNA repeats that are sufficiently abundant and/or clustered above the detection threshold (for example, [91,92]). Of growing relevance, in particular, is the availability to study genomic distribution of satDNAs in silico on advanced genome assemblies (for example, in [62]). 

The total number of satDNAs in the genome, the satellitome, varies significantly among species (Table 1). For example, the satellitome characterization disclosed only one satDNA in the moth *Cydalima perspectalis*, with an abundance of 0.14% [92]. On the other side, among grasshopper insects, 129 satDNAs were detected in the morabine grasshopper [121], while 62 are present in the migratory locust [24]. The ladybird beetle *Hippodamia variegata* (Coleoptera, Coccinellidae) hosts 30 satDNAs that build 15% of its genome [104]. The kissing bug *Rhodnius prolixus*, the principal vector of the Chagas disease, hosts 39 satDNAs that build 8% of the genome [113]. In vertebrates, 164 satDNAs were detected in the fish *Megaleporinus microcephalus,* the most abundant constituting 2.78% of the genome [106], while in the fish *Astyanax lacustris,* none of the 33 detected satDNAs exceeded 0.002% [94]. The same NGS-based clustering approach revealed the largest number of satDNAs, 258, which compose ~28% of the genome of the freshwater crab *Pontastacus leptodactylus*, although 240 out of them were further classified as minisatellites according to their short repeat unit length [109]. The *Triatoma delpontei* satellitome includes 160 satellite DNA families, which, together, constitute significant parts of the insect genome (more than 50%), with the most abundant satDNAs’ contribution being ~18% [118]. The most extensively studied animal genus from the satellitome aspect is *Drosophila*, where 58 species have been inspected and numerous satDNAs characterized (Table 2, [130,131]). 

There is also a great variety of tandem repeats present in plant species. Among them, 91 distinct repeat units grouped as 11 satDNA families compose about 24% of the olive genome [125]. About 25 satDNAs were detected in the satellitomes of the three grass species of the genus *Deschampsia* [123]. In the field bean *Vicia faba*, the NGS sequences and graph-based classification revealed 23 novel satDNAs; however, they represent as little as 0.008–2.7% of the genome [129]. Similarly, the repeatome as a whole builds about 70% of the common oat genome, being mostly composed of TEs (mostly retroelements), while satDNAs are only poorly presented, building only about 2% of genomic DNA [132]. 

Not only a substantial number of (even low-copy) repetitive sequences can be detected by satellitome analysis, as exampled by 164 satDNAs of *Megaleporinus macrocephalus* [106], but such studies can also help in elucidating evolutionary relations among repetitive sequences in related species [94,119,121,124,126,133,134,135,136,137,138,139]. The use of NGS data has also been employed for the studies of evolution of B and sex chromosomes, e.g., to characterize the composition and putative ancestry of B chromosomes in grasshopper species *Rhammatocerus brasiliensis*, *Schistocerca rubiginosa, Xyleus discoideus angulatus, Abracris flavolineata, Eumigus monticola* [93,102,112] or in characid fish *Characidium gomesi* [96]. An understanding of karyotype diversification, genome architecture and sex chromosome evolution was forwarded, for example, in Crambidae moths [92], Triportheidae fishes [140], grasshopper *Ronderosia bergii* [115], sugarcane borer *Diatraea saccharalis* [100], etc. Further questions can be addressed in viewing the satellitome in relation to the repeatome as a whole, especially in the context of the hetero/euchromatin content and distribution.

Even this brief overview shows that the two simple characteristics, number of satDNAs in the satellitome and the total genomic fraction they occupy, are independent and highly variable, depending on various parameters establishing principles that determine organizational patterns of repetitive DNAs in the specific species or the taxonomic group. 

## 5. Comparative Satellitome Analysis: Satellitome and the Library Hypothesis

The satDNA library hypothesis [85] has been proven in the past in many plant and animal species using experimental approaches, mostly based on PCR detection of the low-copy representatives of the highly abundant satDNA from one species in the related taxa [141,142,143,144,145,146], etc. Novel technologies enable in-depth inspection of this hypothesis using in silico analyses on sets of related species. For example, the analysis of 35 satDNAs in three species of fish from the genus *Astyanax* (*A. paranae, A. fasciatus* and *A. bockmanni*) revealed that most of the satDNAs are shared between them, and that they present very similar patterns of chromosomal distribution [95]. Combined low-coverage sequencing and FISH mapping showed that three satDNA families, shared by 10 grasshopper species of the genus *Schistocerca* as part of the satDNA library, follow species phylogeny both in copy number and sequence divergences [147]. In continuation, Pita et al. [119] compared the repetitive genome fraction between Andean and non-Andean lineages of *Triatoma infestans*, characterizing 42 satellite DNA families, 34 present in both lineages, conserved in DNA sequences, but with different amounts present in each lineage. According to the satelitome composition and patterns of chromosomal distribution of satDNAs, Amosova et al. [123] confirmed that genomes of the grass *Deschampsia sukatschewii* and *D. cespitosa* were more closely related in comparison to *D. antarctica*. Similarly, satellitome analysis on Aquitanian mole *Talpa aquitania* and further extension on related species showed that most of the satDNA families are present in the genomes of the other *Talpa* species analyzed, while only some in the genomes of other more distant Talpidae [117]. Following that, comparative analysis of morabine grasshopper genomes revealed that 102 out of 129 satDNA families were shared among the four inspected chromosomal races of the species *Vandiemenella viatica*, and 50 of the shared satDNA families underwent differential proliferation since the recent diversification of the *V. viatica* species complex [121]. In continuation, comparative analysis of the satellitome of the grasshoppers from the Oedipodinae subfamily (*Locusta migratoria* and *Oedaleus decorus*) showed that the 41 satDNA families (20 in *L. migratoria* and 21 in *O. decorus*) belong to 12 ortholog groups and represent the ancestral library. The authors speculate that the remaining 84 families (36 of *L. migratoria* and 37 of *O. decorus*) could represent either remnant satDNAs conserved in only one species or satDNAs arisen de novo during the separate evolution of these species [83]. Following that, evolution of the satellitome following interspecies hybridization of the holocentric root-knot nematodes *Meloidogyne spp.* suggests that the formation of each allopolyploid is accompanied by the addition of a new set of satDNAs, with 39 satDNAs being common for all analyzed species and representing the basic set, characteristic for the 2n genome [108]. Anjos et al. [148] noticed intergenomic variation in the abundance of the satDNA shared between the two *Mahanarva* holocentric insects. This also suggests that the variation in the satDNA amount between species is generally not influenced by the chromosomal architecture (monocentric or holocentric), as will be discussed below. Another example shows that centromere-specific satDNA of the holocentric plant genus *Rhynchospora* has species-specific marks that follow phylogeny in agreement with the predictions of concerted evolution, remaining in the same time among them as components in the library of tandem repeats [149]. 

Aforementioned examples, and many others, show that comparative analysis of satellitomes is opening novel prospects and inputs into satDNA library research. However, it has to be taken into account that the library can be constituted by a subset of sequences detected in the satellitomes, as a certain satellitome may also incorporate a varying number of additional, species-specific satDNAs.

## 6. SatDNA Defying Predictions of the Library Model and the Concerted Evolution

The aforementioned high-throughput studies of repetitive sequences, at the same time, enable re-evaluation of already existing ideas about satDNA evolution. Belyayev et al. [150] pointed out that the satDNA library hypothesis does not address several important questions: how novel satDNAs emerge, how libraries form and survive speciation-related repeatome purification and subsequent concerted evolution and the phenomena of the periodic appearance of novel satDNAs from TEs. In this interpretation, cases of long-term conservation of satellitome elements during evolution would be in accordance with the satDNA library hypothesis, while cases of elimination and/or the emergence of new satDNAs would oppose it [150]. 

In addition, satDNAs were found not to be limited only to closely related species, highlighting the question of their long-term DNA sequence preservation, and of persistence of each of them and of the whole sets in the evolutionary distant taxa (e.g., [151,152,153]). On the one hand, this would potentially broaden the library concept to distantly related species and presume long-term preservation of widespread satDNA sequences derived from the common ancestor, while, on the other hand, the close connection of some of these sequences with TEs affects the conclusions related to their ancestry based only on vertical inheritance and opens the possibility of their horizontal transfer [10]. Such TE-derived or TE-propagated satDNA families may appear in the satellitome in a great number of highly similar copies, creating an illusion of family conservation and/or (artificially) increase the similarity among satellitomes of different species. As such data refute the library hypothesis, Belyayev et al. [150] suggested that it would be more suitable to consider “the library of the mechanisms of origin” instead of “the common satDNA library”.

Concerning the long-term preservation of satDNA sequences, monomers of some satDNAs remain species-indistinctive in the phylogenetic analysis, even after tens or hundreds of My after speciation, as shown in many groups of plant and animal species (for example [103,145,147,151,152,153,154,155,156,157]). The lack of homogenized, species-diagnostic satDNA monomer changes can also be interpreted as an effect of non-concerted evolution, in which sequence variability in satDNA monomers accumulates but mutations do not spread among them because homogenization/fixation are too slow or disabled, as in organisms that reproduce parthenogenetically [158,159]. Our understanding of the causes of this unexpected sequence preservation is still only partial. According to another hypothesis, entire satDNA monomers or their segments can evolve under constraints, thus preserving the once established variability profile of satDNA monomers [9,23,33,66,68,78]. Eventually, as mentioned above, it cannot be completely excluded that the effect of satDNAs preserved among distantly related species is a consequence of horizontal transfer (also see in [10,152]). 

Studying satDNAs in plants of the genus *Chenopodium* showed that non-concerted evolution may result in transformation of the entire satellitome by producing the novel sets of satDNAs in the conversion cycles, thus enabling genomes in which sequence homogenization is suppressed to become a significant source of diversity [160], as anticipated also by Nijman and Lenstra [80]. On the contrary, Smalec et al. [161] reported a satDNA, which maintains conserved sequence and homogenized tandem repeat structure, which results in common, abundant and large blocks of chromatin, homologous among chromosomes within one species and among diverged species, defying, in this way, the molecular drive. They suggest that homogenous heterochromatin may be evolutionarily beneficial in this case by allowing for both intrachromosomal rearrangements and retention of polymorphic variations, contributing to the extremely wide range of ecological adaptations observed for rodents of the *Peromyscus* genus. 

**Figure 1:**
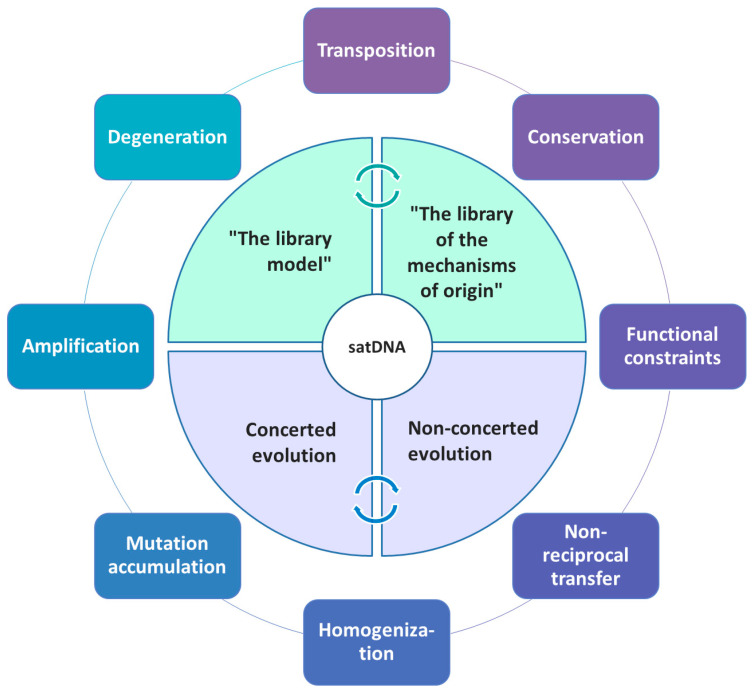
Schematic presentation of different mechanisms and major principles governing/influencing satDNA evolution.

Thus, depending on sequence dynamics in a particular organism or a group of organisms, quite different evolutionary scenarios may occur (Figure 1), ultimately defining the overall satDNA landscape.

## 7. (Slow but Steady) Heterochromatin Mining

Constitutive heterochromatin, as initially defined by Heitz [162], is a chromatin form that remains highly condensed throughout the cell cycle. It is cytologically visible as dense bands on pericentric, telomeric and less frequently on intercalary positions of chromosomes or as chromocenters in interphase nuclei. The DNA sequences dominantly present in constitutive heterochromatin are repetitive, mostly satDNAs. Complex interactions of satDNAs and their transcripts with specific protein components, in combination with unique epigenetic modifications, define specificities in heterochromatin structure and function, such as tightly packed nucleosomes, generally repressive effects on gene expression or the role in maintening the cohesion of sister chromatids [23,163,164].

Despite its functional importance, the content and chromosomal localization of constitutive heterochromatin are highly variable among species, some being heterochromatin-rich and some heterochromatin-poor. For instance, in humans and *Drosophila,* heterochromatin builds 45% and 30% of chromosomes, respectively, while it can form 80% of chromosomes in some plants [16]. The Pacific oyster *Crassostrea gigas* has extremely scarce heterochromatin, notable only on two pairs of chromosomes, in the pericentromeric region of one pair and subtelomeric of another [165]. On the other hand, in the oyster *Crassostrea angulata*, with the possibility of cross-hybridization with *C. gigas*, heterochromatin is abundant and localized at pericentromeric, telomeric and intercalary positions on most of the chromosomes [166]. Similarly, two congeneric species of *Melipona* bees differ significantly in abundance and distribution of heterochromatin on their chromosomes, the difference being, in this case, assigned mostly to the expansion of one satDNA and one TE of those shared among species [107]. At the individual level, heteromorphism of heterochromatin is observed, for instance, as diverse numbers and sizes of C-bands in meiotic bivalents of Heteropteran insects of the genus *Holhymenia* [91,167]. 

Since the introduction of FISH protocols [168], this methodology has been widely used to map DNA sequences on chromosomes. As the signal of highly abundant satDNAs is often strong and coincident with heterochromatic chromosomal segments, it is not surprising that satDNAs are traditionally considered as sequences inevitably associated with heterochromatin, organized as Mb-long arrays of thousands of monomers [7,169]. However, detailed studies of satDNA organization patterns in different species showed that this definition is too narrow (reviewed in [8,10]). For example, satDNAs were detected in euchromatin of *Drosophila* chromosomes, where they are present as dispersed short arrays of repeats highly abundant in the heterochromatin of the same chromosome [170,171]. Mapping of satDNAs identified in the satellitome of the grasshopper *Locusta migratoria* revealed arrays of monomers dispersed along the chromosomes as clusters large enough to be detected by FISH but also as short segments detectable only by bioinformatics analyses of the sequenced genomic DNA. The authors concluded that every satDNA exists in both forms, leading to the suggestion that all genomic sequences repeated in tandem should be considered as satDNAs, regardless of the monomer size and array length, chromatin state or chromosomal localization, as they all follow similar rules [24]. 

In addition to “classical” satDNAs, heterochromatin often accumulates diverse TEs. Some TEs are carrying incorporated tandem repeats, and satDNA arrays can be interrupted with non-repetitive DNA sequences including genes, all in various proportions and interspersion patterns, depending on the species [5,8,16,39]. For example, multiple insertions of TEs into satDNA arrays can be found [172], satDNA repeats can be formed by tandem amplification of a TE or any of its parts [173,174,175,176,177] or by expansion from short internal arrays found within TEs [30,178,179]. SatDNAs in heterochromatin can also intermingle, such as two satDNAs in large domains of pericentromeric heterochromatin of the beetle *Tribolium madens,* that build about 30% of the genome but are arranged in relatively short (up to 70 kb) alternating arrays [180]. By conventional sequencing and mapping, it is particularly difficult to determine the detailed composition, interruption patterns and overall length of long continuous satDNA arrays, because of the prior discussed difficulties in sequencing and assembly. In recent years, the introduction of third-generation sequencing opened the possibility to generate ultra-long reads of genomic DNA, including long segments built of repetitive sequences. Supported by adequate bioinformatics tools, this methodology is a key step forward in deciphering the details of repetitive DNA composition in the previously hardly accessible “dark matter of the genome” [4,6,26]. In some cases, heterochromatin, indeed, dominates Mb-long, only occasionally interrupted, arrays of satDNAs, for instance, in humans [29] or in plants [30]. On the contrary, frequent interruption of satDNA arrays by retrotransposon element has been documented in the heterochromatin of end-to-end assembled maize chromosomes [181]. Long-read sequencing technologies supported by assembly-free methods revealed, in the grass pea *Lathyrus sativus,* only 2 out of 11 major satDNAs in the typical form of long arrays associated with centromeric chromatin or subtelomeric heterochromatin, while the rest represent amplified tandem repeats of a retrotransposon origin accumulated in the (peri)centromeric regions [30]. Further on, a detailed view on the organization pattern of DNA sequences in dispersed heterochromatic bands of the holocentric plant *Cuscuta europea* showed a complex arrangement of up to 10 kb-long arrays of a highly amplified satDNA and other repetitive elements [31]. In *Chorthippus parallelus,* the high number of tandem repeats with sequence homology to TEs exist, and the authors suggest that some of them might actually be tandem repeat-carrying TEs and that their interspersed distribution could be the reason for the inability for visualization by FISH [98]. Short arrays of satellite repeats are characteristic for the oyster *Crassostrea gigas*, dominantly located within the TEs of the Helitron superfamily [61]. In addition, TEs themselves can be abundant components accumulated in heterochromatin, as in *Drosophila* (reviewed in [16,147]). Further, as evidenced by high-throughput next-generation sequencing of H3K9me3/2-associated sequences, heterochromatin of the Pacific oyster *C. gigas*, [182] or *Beta vulgaris* [183] is dominantly composed of various TEs. However, the relationship between the heterochromatin size and the composition and content of repetitive sequences in a genome is complex, and in this moment, it is only partially understood. 

## 8. In and Out of Heterochromatin

**Figure 2:**
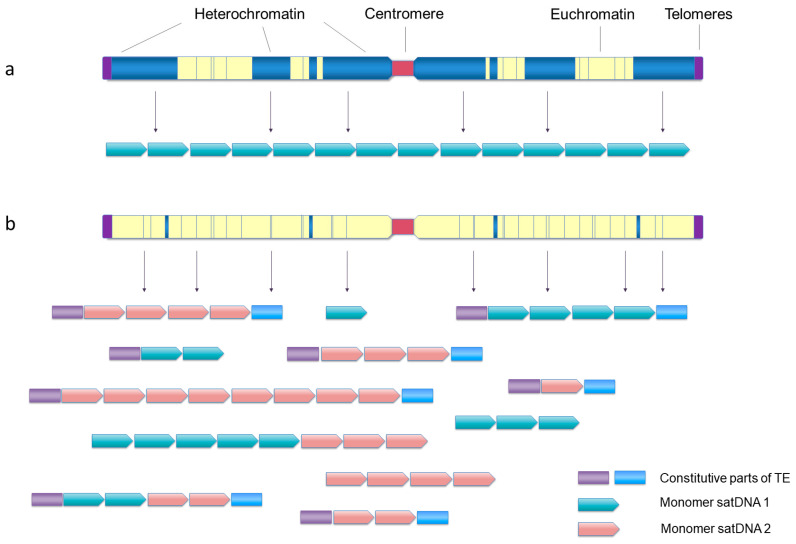
Two significantly different patterns of satDNA organization at the genome level. (**a**) satDNAs occupying large pericentromeric, subtelomeric and interstitial blocks of heterochromatin, with sporadic short arrays or single monomers situated in the euchromatin. (**b**) satDNAs organized in short arrays, highly dispersed throughout the chromosomes without any significant clustering, frequently found associated with TEs or their parts or short arrays of other satDNAs.

Although our comprehension about genome-wide dispersal of satDNAs is still limited to a small number of species, it seems that such distribution can be a rule rather than an exception. The existence of dispersed monomers and/or short arrays is predicted as an intermediate stage in the hypothesis about the onset of large heterochromatin-associated arrays [24,65]. However, when analyzing “out of heterochromatin” copies, it is difficult to predict the direction of the spread of satDNA monomers as many factors are probably involved in the generation of this pattern (i.e., dominantly localized and sporadically dispersed). In the case of human alpha satDNA, major clusters, located in the pericentromeric regions, were indicated as sources of euchromatic copies, and the suggested spread is thought to be driven by a rolling-circle mechanism [184]. In the red-flour beetle *Tribolium castaneum*, >30% of the genome is composed of a satDNA family localized by FISH in large blocks of pericentromeric heterochromatin [185]. However, a small fraction of monomers of this and of several other heterochromatin-residing satDNAs could be detected within the assembled euchromatic genome fraction, often in the relative vicinity of genes ([186], Figure 2a). It was postulated that euchromatic copies of pericentromeric satDNAs in *T. castaneum* are functionally significant in modulating chromatin and the expression of nearby genes under stress conditions [46]. Similarly, euchromatic copies of *Drosophila melanogaster* 1.688 satDNA are mostly positioned in the vicinity of genes as short arrays, mostly of up to six repeats, and have a probable role in the regulation of gene expression [171].

Opposing the established paradigm of clustering in heterochromatin, some satDNA families of the red-flour beetle *T. castaneum* were detected only on the euchromatic chromosomal segments, although their presence in heterochromatin could not be completely excluded [187]. Similarly, several satDNA families were detected on the euchromatic regions of the autosomes and the X chromosome of the hemipteran insect *Triatoma infestans* [119]. The complex genomic distribution of satDNAs is described in the red palm weevil, *Rhynchophorus ferrugineus* (Coleoptera), a rapidly spreading invasive species causing severe damage to palm trees. Its satellitome builds 25% of the genome, and abundant families were found to be dominantly deposited in euchromatin, although they are also distributed in the pericentromeric heterochromatin of all chromosomes or on specific chromosomes only. Interestingly, the copy number of some satDNA families is increased in populations that invaded new habitats most recently [114].

As already commented, short arrays of <10 monomers (sometimes called satDNA-like) are often dispersed in euchromatin as constitutional components of TEs in diverse species (for example, [57,59,60,171,178,179,188,189]). They may represent the sources of “classical” satDNA arrays by “filling” the heterochromatic domains, and, at the same time, may also be a cause of dispersal of tandem arrays. The extensive association of tandem repeats and TEs of the Helitron/Helentron superfamily and shuffling of arrays is a probable cause of the exceptional satDNA genomic landscape in the Pacific oyster *C. gigas* [61]. It is characterized by an unusual lack of clustering of relatively short arrays or single monomers of all satDNAs. Instead, most of them are uniformly dispersed as TE-associated or standalone repeats along the entire chromosomal arms of all chromosomes ([62], Figure 2b). While *C. gigas* is a species with monocentric chromosomes (localized centromere function), such dominantly dispersed organization of satDNA arrays along the chromosomes and diversity in heterochromatin—euchromatin localization—are particularly evident in species with holocentric centromeres, either plant or animal. Comparisons of high-quality genome assemblies of closely related species with repeat-based centromeres, the monocentric *Juncus effusus* and the holocentric beak-sedges *Rhynchospora* spp. showed rebuilding of heterochromatin compartments and redistribution of satDNAs, thus changing the genome architecture in transition from monocentricity to holocentricity [190]. In the case of *Rhynchospora*, the holocentromeres are mostly composed of short arrays, 20–25 kb, of a satDNA named *Tyba*, uniformly distributed along the chromosomes and specifically co-localizing with the centromere-determinant protein CenH3 [191]. The authors suggest that the *Tyba* satDNA family is widely distributed and conserved in about 70 examined *Rhynchospora* species separated for about 30 My because of its sequence-dependent role in the centromeric function [149]. Some *Tyba* repeats are found to be linked with a Helitron TE, which probably drives their dispersal [190]. Conservation because of the sequence-dependent role in the centromeric function was also concluded for the satDNA of the holocentric *Meloidogyne* root-knot nematode species [192]. 

Satellitome research revealed the distribution of euchromatin- and heterochromatin-dominant satDNAs in several other species with holocentric chromosomes. In the two evolutionary lineages of the hemipteran insect *Triatoma infestans*, 7 out of 11 FISH-localized satDNAs were unexpectedly detected on euchromatic regions of the autosomes and the X chromosome. Only one of the euchromatic satDNAs is in its high abundance comparable with heterochromatic ones, while the rest are low abundant, and the genomic variations between the lineages are mostly due to differences in abundance of satDNAs associated with heterochromatin [119]. Widening of the comparative studies by including congeneric species *T. delpontei* indicated a high level of heterochromatin-euchromatin satDNA localization shuffling during speciation. While the *T. delpontei* genome harbors numerous satDNAs (160, >50% of the genome), heterochromatin is formed mainly by just four. Two of these satDNAs are also present in the heterochromatin of *T. infestans,* while the other two were located in the euchromatin. Vice versa, there were also satDNAs located in the euchromatin in *T. delpontei* that are part of *T. infestans* heterochromatin. Noteworthily, for satDNAs located mainly in the heterochromatin of *T. delpontei*, less intense hybridization signals were also observed in the autosomal euchromatic regions [118], organizational pattern presented also in Figure 2a. In another holocentric species, the kissing bug *Rhodnius prolixus*, heterochromatic is only the entire Y chromosome, while mapped satDNAs revealed dispersed FISH signals in the euchromatin of all chromosomes, despite the lack of detectable constitutive heterochromatin [113].

Analyses of sequenced genomes and comparative satellitomics established satDNAs or satDNA-like tandem repeats as the common euchromatin component. They can exist as short arrays sharing the nucleotide sequence with the (major) satDNA(s) located in the heterochromatin (Figure 2a) or as tandem repeats dominantly located in euchromatin, as exampled by the Pacific oyster *C. gigas* (Figure 2b). Euchromatin-dominant satDNAs have essential structural, organizational and evolutionary features similar to their counterparts in heterochromatin. The most significant difference could be in rates of sequence homogenization, which is less efficient among distantly located and shorter arrays than in clustered and longer ones (reviewed also in [66]), as reported in comparisons of euchromatic and heterochromatic arrays of *Drosophila melanogaster* 1.688 repeats [171] or in comparisons of dispersed satDNA arrays in the species of the Hemipteran genus *Mahanarva* [148]. 

## 9. Conclusions

In conclusion, the advent of novel strategies in the analysis of repetitive DNA sequences followed by the burst of studied non-model organisms showed significant differences in organizational principles of satDNAs and their localization on the chromosomes. Recent studies have shown a large diversity in satellitomes, from only 1 to over 200 satDNAs, located not only in heterochromatin but also in euchromatin, regardless of the centromere organization and overall chromosomal architecture. Presently, it seems that each of the characteristics related to sequences repeated in tandem (their number, abundance, organization, distribution, heterochromatin/euchromatin localization) represent features independent of each other. Additionally, comparative satellitome studies brought new details, questioning the established views on satDNA evolution. However, it is still too early to make some general conclusions, because of diversity in the inspected systems, and as detailed studies of satDNA arrays in the genome are still scarce and often fragmentary, focused only on some aspects of satDNA landscapes. It would also be of use to provide a more comparable view on satellitomes by making inputs and outputs of analyses comparable wherever possible (e.g., the genome fraction occupied by satDNAs, the detection level, chromosomal mapping, etc.). Ultimately, the need to introduce new species as model systems is rising, as significant and extremely relevant information arises from different systems and contributes greatly to the research area of repetitive DNA biology.

## Figures and Tables

**Table 1:** Examples of diversity in number and genome abundances of satDNAs across a spectrum of animal and plant species.

Species	Number of satDNAs	% of the Genome	Most Abundant satDNA (%)	Reference
**animals**				
*Abracris flavolineata*	53	4.52	1.73	[93]
*Astyanax lacustris*	33	-	0.001	[94]
*Astyanax paranae*	45	8.39	6.30	[95]
*Characidium gomesi*	59	3.98	0.58	[96]
*Choloepus didactylus*	2	13.62	13	[97]
*Choloepus hoffmanni*	2	2.83	2.6	[97]
*Chorthippus parallelus*	110	-	1	[98]
*Colossoma macropomum*	46	<1	0.013	[99]
*Crassostrea gigas*	52	6.33	1.29	[62]
*Cydalima perspectalis*	1	0.14	0.14	[92]
*Diatraea postlineella*	2	0.06	0.04	[92]
*Diatraea saccharalis*	14	0.215	0.057	[100]
*Eneoptera surinamensis*	45	14	1.41	[101]
*Eumigus monticola*	27	1.91	0.55	[102]
*Gryllus assimilis*	13	4	1.35	[103]
*Hippodamia variegata*	30	14.93	9.37	[104]
*Locusta migratoria*	62	2.39	0.98	[24]
*Megaleporinus elongatus*	140	~5	0.48	[105]
*Megaleporinus macrocephalus*	164	13.47	2.78	[106]
*Melipona quadrifasciata*	13	2.83	0.94	[107]
*Melipona scutellaris*	13	38.4	38.2	[107]
*Meloidogyne arenaria*	81	5.07	0.31	[108]
*Meloidogyne floridensis*	38	1.55	0.14	[108]
*Meloidogyne incognita*	56	3.52	0.24	[108]
*Meloidogyne javanica*	73	4.27	0.21	[108]
*Oedaleus decorus*	58	2.53	0.62	[83]
*Ostrinia nubilalis*	4	0.27	0.15	[92]
*Piaractus mesopotamicus*	30	<1	0.009	[99]
*Pontastacus leptodactylus*	258	27.57	10.91	[109]
*Proceratophrys boiei*	28	15.87	8.0	[110]
*Psalidodon bockmanni*	50	-	0.016	[94]
*Psalidodon fasciatus*	57	-	0.091	[94]
*Pyrgomorpha conica*	76	9.4	5.66	[111]
*Rhammatocerus brasiliensis*	12	1.49	0.76	[112]
*Rhodnius prolixus*	39	8	2.13	[113]
*Rhynchophorus ferrugineus*	112	25	20.4	[114]
*Ronderosia bergii*	53	2.44	0.43	[115]
*Schistocerca rubiginosa*	9	2.17	0.73	[112]
*Spodoptera frugiperda*	7	0.65	0.23	[116]
*Talpa aquitania*	15	1.24	0.55	[117]
*Triatoma delpontei*	160	18.15	53.92	[118]
*Triatoma infestans*	42	25	10.04	[119]
*Trigona hyalinata*	8	16.56	13.77	[120]
*Vandiemenella viatica*	129	-	1.48	[121]
*Xyleus discoideus angulatus*	18	2.32	0.62	[112]
**plants**				
*Aegilops crassa*	19	-	0.95	[122]
*Deschampsia antarctica*	20	2.07	0.21	[123]
*Deschampsia cespitosa*	27	2.85	0.69	[123]
*Deschampsia sukatschewii*	21	1.61	0.22	[123]
*Larix decidua*	5	3.2	1.28	[124]
*Larix kaempferi*	4	2.0	0.81	[124]
*Olea europaea cuspidata*	11	50.43	22.95	[125]
*Olea europaea europaea*	11	23.89	7.89	[125]
*Olea europaea guanchica*	11	23.35	9.23	[125]
*Olea exasperata*	11	26.43	15.74	[125]
*Olea paniculata*	11	1.93	0.79	[125]
*Passiflora cincinnata*	2	-	0.10	[126]
*Passiflora edulis*	2	0.22	0.16	[127]
*Passiflora organensis*	37	-	3.50	[126]
*Passiflora quadrangularis*	6	-	0.13	[126]
*Thinopyrum bessarabicum*	12	-	1.39	[122]
*Vandenboschia speciosa*	11	0.43	0.08	[128]
*Vicia faba*	23	-	2.72	[129]

**Table 2:** Genome contributions of satDNAs detected in the species of the *Drosophila* genus.

Species	Number of satDNAs	% of the Genome	Most Abundant satDNA (%)	Reference
*Drosophila affinis*	4	2.07	0.911	[130]
*Drosophila albomicans*	6	38.8	36.946	
*Drosophila americana*	8	19.75	9.501	
*Drosophila ananassae*	6	3.68	1.41	
*Drosophila arizonae*	2	0.54	0.348	
*Drosophila biarmipes*	7	1.27	0.31	
*Drosophila bipectinata*	7	4.72	1.31	
*Drosophila burlai*	5	3.12	1.86	
*Drosophila busckii*	5	1.1	0.503	
*Drosophila buzzatii*	2	1.9	1.71	
*Drosophila elegans*	6	4.01	1.39	
*Drosophila erecta*	3	1.62	1.138	
*Drosophila eugracilis*	3	10.89	5.691	
*Drosophila ficusphila*	2	1.76	1.682	
*Drosophila hydei*	5	2.16	0.733	
*Drosophila kikkawai*	3	4.85	2.493	
*Drosophila leontia*	6	1.81	1.34	
*Drosophila malerkotliana*	6	6.04	2.40	
*Drosophila mauritiana*	7	4.86	3.58	
*Drosophila melanogaster*	5	6.6	1.75	
*Drosophila mojavensis baja*	2	1.76	1.06	
*Drosophila mojavensis wrigley*	2	2.49	1.63	
*Drosophila montana*	6	27.41	19.70	
*Drosophila nasuta*	7	33.93	32.68	
*Drosophila novamexicana*	7	6.82	3.03	
*Drosophila orena*	3	12.31	10.40	
*Drosophila persimilis*	4	5.87	5.20	
*Drosophila pseudoobscura*	4	5.48	1.93	
*Drosophila rhopaloa*	3	4.67	4.34	
*Drosophila santomea*	7	2.7	1.82	
*Drosophila sechellia*	7	7.72	6.04	
*Drosophila seriema*	4	2.9	1.93	
*Drosophila simulans*	8	4.53	3.18	
*Drosophila subobscura*	6	1.4	0.72	
*Drosophila takahashii*	5	3.95	0.98	
*Drosophila teissieri*	4	2.09	0.57	
*Drosophila virilis*	7	21.63	15.9	
*Drosophila yakuba*	5	2.83	1.18	
*Drosophila asahinai*	3	6.87	6.30	[131]
*Drosophila auraria*	2	4.72	4.69	
*Drosophila bakoue*	9	1.88	0.36	
*Drosophila birchii*	8	5.01	3.23	
*Drosophila bocki*	7	1.89	0.41	
*Drosophila bunnanda*	14	15.69	5.98	
*Drosophila burlai*	7	7.67	3.12	
*Drosophila jambulina*	6	12.24	5.87	
*Drosophila kanapiae*	4	1.98	0.78	
*Drosophila lacteicornis*	3	7.20	6.62	
*Drosophila leontia*	4	1.99	0.90	
*Drosophila mayri*	13	17.77	9.54	
*Drosophila nikananu*	3	8.33	2.80	
*Drosophila pectinifera*	10	21.65	15.56	
*Drosophila punjabiensis*	6	2.12	0.94	
*Drosophila rufa*	3	5.78	5.46	
*Drosophila seguyi*	12	10.73	4.58	
*Drosophila serrata*	6	14.50	10.46	
*Drosophila tani*	4	5.99	4.57	
*Drosophila triauraria*	3	5.70	5.43	
*Drosophila truncata*	5	8.92	4.17	
*Drosophila vulcana*	3	5.97	5.59	
*Drosophila watanabei*	3	1.40	0.74	

## Data Availability

Not applicable.
